# The psychometric properties of the barriers to insulin treatment questionnaire in Chinese patients with type 2 diabetes mellitus using insulin

**DOI:** 10.3389/fendo.2023.1192108

**Published:** 2023-08-15

**Authors:** Min Ma, Xiquan Ma, Jingzhi Chang, Feiyan Yin, Sha Ma, Yuan Zhang, Zhidao Shi

**Affiliations:** ^1^ Psychological Counseling and Therapy Center, Wuhan Mental Health Center, Wuhan Hospital for Psychotherapy, Wuhan, China; ^2^ Department of Developmental and Behavioral Pediatrics, Shanghai Children’s Medical Center, School of Medicine, Shanghai Jiao Tong University, Shanghai, China; ^3^ Department of Public Health Sciences, Faculty of Medicine, University of Southampton, Southampton, United Kingdom; ^4^ Clinical Research Center for Mental Disorders, Shanghai Pudong New Area Mental Health Center, School of Medicine, Tongji University, Shanghai, China

**Keywords:** type 2 diabetes mellitus, barriers to insulin treatment, psychological resistance to insulin therapy, adherence, scale revision, reliability, validity

## Abstract

**Aim:**

The objective of this study was to translate the Barriers to Insulin Treatment Questionnaire (BIT) into Chinese and test its psychometric properties in middle-aged and elderly type 2 diabetes mellitus (T2D) patients using insulin in the Han people of urban China.

**Methods:**

We established the Barriers to Insulin Treatment Questionnaire in Chinese (BIT-C). We selected 296 patients with T2D for testing BIT-C's the reliability and validity, of which 120 patients were retested four weeks later. Another 200 patients with T2D were selected to perform the confirmatory factor analysis (CFA).

**Results:**

The final BIT-C consisted of 11 items (BIT-C-11) and four factors. The explained variances of the BIT-C-11 and its four factors were 90.153%, 51.308%, 18.810%, 10.863%, and 9.173%. CFA validated that the four-factor model fit with the data of the BIT-C-11. Standardized factor loadings ranged between 0.77 and 0.90. The Cronbach’s α coefficients of the BIT-C-11 and its four factors were 0.903, 0.952, 0.927, 0.938, and 0.917. Correlation analysis was performed between the BIT-C-11 and General Adherence Scale in Chinese (GAS-C) to calculate the criterion-related validity (r = 0.598, p < 0.001). The correlation coefficient r of the BIT-C-11’s test–retest reliability was 0.810 (p < 0.001).

**Conclusion:**

The BIT-C-11 has good reliability and validity. It can be used for psychological resistance to insulin therapy studies of middle-aged and elderly patients with T2D using insulin in the Han people of Chinese cities.

## 1 Introduction

If non-insulin medication has failed, type 2 diabetes mellitus (T2D) patients may need insulin injections to control hyperglycemia to recommended levels (1, 2). However, studies in recent years have found poor adherence in patients with T2D who use insulin to treat their diabetes (3, 4).

A 2017 study found that the insulin adherence and persistence of patients with T2D in China are generally poor. Only 53% of patients with T2D persisted with insulin therapy until 12 months. After 1 year of insulin injections, only 30.9% of patients with T2D had a medication possession rate (MPR) ≥0.8 (5). Psychological factors such as negative beliefs about insulin therapy are the most common reasons for these patients’ poor adherence to insulin therapy (6, 7). Psychological insulin resistance (PIR) is a barrier for providers and patients in starting and maintaining insulin therapy (8). The patient’s psychological resistance to insulin therapy can result in poor glycemic control, damaging their health and burdening their families and society (9, 10). China has many patients with T2D, and many need insulin to control their blood sugar (11, 12). Improving the adherence of these patients with T2D who require long-term insulin therapy is an urgent challenge for the prevention and control of T2D in China. Regarding population health, it may be more effective to focus efforts on those who are least likely to adhere or those with poorly controlled diseases (13). Therefore, there is an urgent need to investigate psychological resistance to insulin therapy in patients with T2D in China. However, no standardized research tools can quantitatively assess psychological resistance to insulin therapy in patients with T2D in China. The Barriers to Insulin Treatment Questionnaire (BIT) is a valuable tool for studying psychological resistance to insulin therapy in patients with T2D, which Petrak et al. (6) developed. This scale has been widely used (14, 15). So, we decided to revise the Barriers to Insulin Treatment Questionnaire in Chinese (BIT-C) and select middle-aged and elderly Chinese patients with T2D who were on insulin therapy as the research objects to evaluate the reliability and validity of the BIT-C.

## 2 Materials and methods

### 2.1 Participants

This study has two parts:

Study I: translating the BIT into Chinese and conducting an exploratory factor analysis (EFA) on it;
Study II: confirmatory factor analysis (CFA) of the Chinese version of the BIT.

This study was conducted following the Declaration of Helsinki and was approved by the Shanghai Pudong New Area Mental Health Center (approval number: 2017009). It was conducted from May 2018 to December 2020 in the diabetes wards of several general hospitals in Haicheng City in northeast China.

The inclusion criteria of the study subjects were as follows:

- Patients who meet the WHO diagnostic criteria for T2D,
- currently on insulin therapy,
- aged 45–74 years,
- Han Chinese who had been continuously residing at the survey site for at least 5 years at the time of the survey,
- voluntary participation.

Subjects will not be included in our study if they match the following exclusion criteria:

- those who were seriously ill and unable to complete the study,
- those who had a disturbance of consciousness,
- those suffering from various severe mental illnesses who cannot complete the study.

### 2.2 Instruments

#### 2.2.1 General information questionnaire

Demographic and medical data (glycosylated hemoglobin level, insulin use, diabetes duration) were collected by self-report.

#### 2.2.2 BIT

Petrak et al. (6) developed the BIT, which measures psychological resistance to insulin treatment in patients with T2D. The BIT includes 14 items, a total sum score, and the following five factors: Factor 1: fear of injection and self-testing (items 1–3); Factor 2: expectations regarding positive insulin-related outcomes (items 4–6; they were reverse coded); Factor 3: expected hardship from insulin treatment (items 7–9); Factor 4: stigmatization by insulin injections (items 10–12); Factor 5: fear of hypoglycemia (items 13 and 14) (**Table 1**). The response format of the BIT is a 10-point Likert scale, ranging from “totally disagree” [1] to “totally agree” [10]. The BIT’s Cronbach’s α for the five subscales ranged from 0.62 to 0.85, and the BIT’s α for the total sum score was 0.78 (6). It will be revised in Chinese in this study.

**Table 1:** Summary of item analysis of the BIT-C-14.

Factors of the BIT	Items of the BIT	R	K/D
1: fear of injection and self-testing	1: I am afraid of the pain when injecting insulin.	0.610**	keep
	2: Besides the pain, I am just afraid of injections.	0.670**	keep
	3: I am afraid of the pain during regular blood-sugar checks.	0.656**	keep
2: expectations regarding positive insulin-related outcomes	4: Insulin works better than pills.	0.173**	**delete**
	5: People who get insulin feel better.	0.246**	**delete**
	6: Insulin can reliably prevent long-term complications due to diabetes.	0.257**	**delete**
3: expected hardship from insulin treatment	7: I just don’t have enough time for regular doses of insulin.	0.823**	keep
	8: I can’t pay as close attention to my diet as insulin treatment requires.	0.745**	keep
	9: I can’t organize my day as carefully as insulin treatment requires.	0.771**	keep
4: stigmatization by insulin injections	10: Injections in public are embarrassing to me. Pills are more discreet.	0.614**	keep
	11: Regular insulin treatment causes feelings of dependence.	0.617**	keep
	12: When people inject insulin, it makes them feel like drug addicts.	0.618**	keep
5: fear of hypoglycemia	13: An insulin overdose can lead to extremely low blood-sugar levels (“hypoglycemia”). I am afraid of the unpleasant accompanying symptoms.	0.564**	keep
	14: An insulin overdose can lead to extremely low blood-sugar levels (“hypoglycemia”). I have concerns about possible permanent damage to my health.	0.589**	keep

**Correlation is significant at the 0.01 level.

BIT, Barriers to Insulin Treatment Questionnaire; BIT-C-14, Chinese version of the BIT questionnaire with 14 items; R, correlation coefficient of each item to the total score of the BIT-C-14; K/D, kept or deleted.

#### 2.2.3 GAS-C

The General Adherence Scale (GAS) was developed by DiMatteo and Hays and is used to assess the general tendency of patients with chronic diseases to adhere to their physicians’ recommendations during the past 4 weeks (16, 17). Shi revised the General Adherence Scale in Chinese (GAS-C), which can be applied to the general adherence study of middle-aged and elderly patients with T2D in China. Consistent with the GAS, the GAS-C has five items and is one-dimensional. The Cronbach’s α reliability coefficient of the GAS-C was 0.942 (18). In this study, the GAS-C was used to assess the criterion-related validity of the BIT-C.

### 2.3 Translation and adaptation of the scale

After obtaining the developer’s permission, we integrated the cross-cultural approach to translate and adapt the BIT into Chinese (19, 20). The translation and adaptation stages of the BIT are as follows:

#### 2.3.1 Forward translation

Two bilingual translators translated the BIT into Chinese separately. One translator is a teacher in the Department of English, and the other is a research group member.

#### 2.3.2 Synthesis of the translations

Team members and two translators analyzed and compared the two drafts resulting from StageI, producing one common translation draft of the BIT.

#### 2.3.3 Back translation

Two other translators with no medical background translated the Chinese BIT draft into English separately to produce two back-translation English BIT scales.

#### 2.3.4 Expert committee review

The expert committees involved two linguists, one epidemiologist, four translators (forward and back translators), and research team members. They analyzed and compared the BIT, two forward translation versions, one common translation draft, and two back-translation BIT to finalize the initial Chinese version of the BIT. After discussion, the expert committee concluded that this initial Chinese version of the BIT is equivalent to the original version of the BIT in terms of semantics, idiomatic expression, experience, and concepts.

#### 2.3.5 Pretesting and cognitive interviews

Fifteen patients with T2D who met the research criteria were asked to fill in the initial Chinese version of the BIT. Patients all filled out the questionnaire without any problems. Next, we conducted a cognitive interview with these patients to examine the comprehensibility of the questionnaire (21). All patients reported that they could understand each questionnaire item without ambiguity.

#### 2.3.6 Establishment of the final Chinese version of the BIT (BIT-C-14)

After discussion, we decided to use the 14-item initial Chinese version of the BIT as the final Chinese version of the BIT (BIT-C-14).

### 2.4 Data collection

Data were collected the day before the study subjects were discharged from the hospital. Patients who met the subject criteria and agreed to participate in this study were included. Before filling out the questionnaire, subjects were asked to sign an informed consent form. If the subjects have poor eyesight or cannot read or write, the investigator will read the questionnaire aloud and fill out the items according to their true feelings. After the subjects completed the questionnaire, investigators asked if they would like to participate in the retest. We randomly selected 120 subjects who agreed to be retested. They would be investigated again when they returned to the outpatient clinic for physician follow-up at week 4 after discharge.

### 2.5 Statistical analysis

We conducted the statistical analysis using SPSS 23.0. The sociodemographic information of the subjects was described by mean and standard deviation, frequency, and percentage. Continuous variables were expressed by mean ± standard deviation (SD). Counts and percentages are used to indicate categorical variables. p < 0.05 means a statistically significant difference. AMOS 23.0 was used in the CFA.

#### 2.5.1 Item analysis

Using the homogeneity test to analyze items, those items with a low correlation with the total score on the BIT-C-14 were removed. The removal criteria are the value of the Pearson correlation coefficient r < 0.4 or the significant difference test p ≥ 0.05 (22). If deleting an item may significantly increase the Cronbach’s α value of the scale, it means that the item is not homogeneous with the rest of the items, and the item will be removed from the scale (21). For the EFA, those items with communalities <0.2 would be removed (23).

#### 2.5.2 Validity analysis

We analyzed the scale’s content validity, construct validity, and criterion-related validity.

Six diabetologists evaluated the scale’s content validity based on the item analysis results. They rated the degree of correlation between the content of each item and the evaluation purpose for that item. Content validity was judged by the item-level content validity index (I-CVI) and content validity index (S-CVI/Ave). We would retain those items with I-CVI ≥0.78; if the S-CVI/Ave ≥0.9, the scale-level content validity is acceptable (24, 25). Otherwise, the unqualified items should be deleted or modified and reevaluated until they meet the criteria.

We performed the EFA to test the scale’s construct validity. If the Kaiser-Meyer-Olkin measure of sampling adequacy (KMO) ≥0.70 and the difference of the Bartlett’s test had statistical significance (p < 0.05), the scale was suitable for factor analysis. If an item’s measure of sampling adequacy (MSA) is <0.5, the item is unsuitable for factor analysis and will be deleted (26, 27). We chose principal component analysis (PCA) combined with the Varimax orthogonal rotation method to analyze the data. The following criteria were used to determine the number of factors. 1) Kaiser’s principle of eigenvalues >1 to extract factors (28). 2) The factor contains at least two items with loadings >0.4 (29). 3) Items with cross-loading >0.75 were deleted (29). The scree test will assist us in judging the results of the PCA. Ultimately, the EFA’s results and the original BIT’s theoretical structure will guide us in determining the final version of the scale (28). We used the following criteria to assess the goodness of the CFA model: the ratio of chi-square to degrees of freedom (CMIN/df) <5; standardized root mean square residual (SRMR) <0.05; root mean square error of approximation (RMSEA) <0.08; comparative fit index (CFI), the goodness of fit index (GFI), and Tucker–Lewis index (TLI) value >0.9 (30, 31).

We used the GAS-C as a validity criterion to analyze the scale’s criterion-related validity. The criterion-related validity is acceptable if the Pearson correlation coefficient r ≥ 0.4 and is statistically significant (32).

#### 2.5.3 Reliability analysis

We evaluated the scale’s reliability. Its internal consistency reliability is appropriate if Cronbach’s α ≥ 0.70 (21). The intraclass correlation coefficient (ICC) between 0.6 and 0.74 is good, and ≥0.75 is excellent (33). We assessed the test–retest reliability also. The Pearson correlation coefficient r of test–retest reliability ≥0.7 is acceptable (34).

#### 2.5.4 Ceiling effect and floor effect

We evaluated the ceiling and floor effects of the data. A ceiling or floor effect exists if more than 15% of respondents achieve an item’s maximum or minimum score, meaning a response bias occurred in the data (35).

## 3 Results

Data from 496 patients with T2D were collected, from whom 296 patients with T2D were randomly selected as subjects for the item analysis, reliability analysis, and validity analysis of the BIT-C-14—the remaining 200 patients with T2D as subjects for the CFA. There were no missing data. Descriptive statistics for participants’ socioeconomic, medical, and psychological variables in Study I and Study II are provided in **Table 2**.

**Table 2:** General characteristics of the participants in samples A and B.

Characteristics	Sample A	Sample B
**n**	296	200
**Age(years)**	63.51 ± 7.88	62.05 ± 8.60
**Sex**		
Male (%)	53.04 (157/296)	52.50 (105/200)
Female (%)	46.96(139/296)	47.50 (95/200)
**Education levels(years)**	9.45 ± 2.98	9.51 ± 2.81
**Duration of diagnosis(month)**	87.16 ± 67.92	86.08 ± 64.42
**HbA1c**		
Mean (SD), %	8.7(1.8)	8.6(1.6)
Mean (SD), mmol/mol	72(20)	70(18)
**Total score of the BIT-C-14**	70.91 ± 22.86	–
**Total score of the GAS** **Total score of the BIT-C-11**	19.83 ± 5.05 58.61 + 22.50	- 56.46 + 17.30

Data are means ± SD or percentages.

n, the sample size; HbA1c, hemoglobin A1c; BIT-C-14, Chinese version of the BIT questionnaire with 14 items; GAS, General Adherence Scale; BIT-C-11, Chinese version of the BIT questionnaire with 11 items; Sample A, participants of the exploratory factor analysis; Sample B, participants of the confirmatory factor analysis.

### 3.1 Item analysis

The Pearson correlation coefficients for the BIT-C-14’s items 4–6 with the BIT-C-14’s total score were all <0.4. The poor correlation means that these three items are not homogeneous with the remaining 11 items of the BIT-C-14 (22). So, we deleted them and obtained a BIT-C scale with the remaining 11 items (BIT-C-11) (**Table 1**). No items with communalities were <0.2 (21, 23) (**Table 3**). Removing an item from the BIT-C-11 would not increase its Cronbach’s α value (**Table 4**). All 11 items in the BIT-C-11 were retained.

**Table 3:** Summary of the BIT-C-11’s exploratory factor analysis.

Items of the BIT-C-11	Component				MSA	IC
	1	2	3	4		
Factor 1: “fear of injection and self-testing”						
1. I am afraid of the pain when injecting insulin.	0.896				0.872	0.893
2. Besides the pain, I am afraid of injections.	0.905				0.856	0.914
3. I am afraid of the pain during regular blood-sugar checks.	0.922				0.810	0.934
Factor 2: “expected hardship from insulin treatment”						
4. I just don’t have enough time for regular doses of insulin.			0.762		0.908	0.841
5. I can’t pay as close attention to my diet as insulin treatment requires.			0.889		0.842	0.890
6. I can’t organize my day as carefully as insulin treatment requires.			0.874		0.823	0.917
Factor 3: “stigmatization by insulin injections”						
7. Injections in public are embarrassing to me. Pills are more discreet.		0.903			0.857	0.871
8. Regular insulin treatment causes feelings of dependence.		0.913			0.811	0.903
9. When people inject insulin, it makes them feel like drug addicts.		0.922			0.831	0.901
Factor 4: “fear of hypoglycemia”						
10. An insulin overdose can lead to extremely low blood-sugar levels (“hypoglycemia”). I am afraid of the unpleasant accompanying symptoms.				0.921	0.734	0.933
11. An insulin overdose can lead to extremely low blood-sugar levels (“hypoglycemia”). I have concerns about possible permanent damage to my health.				0.885	0.764	0.920
**Eigenvalue**	5.644	2.069	1.195	1.009		
**Variance explained (%)**	51.308	18.810	10.863	9.173		
**Total variance explained (%)**	90.153					

Extraction method: Principal component analysis (Kaiser’s eigenvalue >1); Four components extracted; Factor Loadings > 0.40 are reported.

BIT-C-11, Chinese version of the BIT questionnaire with 11 items; MSA, measures of sampling adequacy; IC, item’s communalities.

**Table 4:** Reliability of the BIT-C-11.

Item Number	Means ± SD	α
1	5.03 ± 2.59	0.895
2	5.16 ± 2.82	0.893
3	4.92 ± 2.77	0.894
4	4.79 ± 2.95	0.886
5	5.63 ± 2.95	0.893
6	5.16 ± 2.99	0.889
7	5.40 ± 2.89	0.899
8	5.08 ± 2.93	0.897
9	5.10 ± 3.01	0.898
10	6.29 ± 2.84	0.900
11	6.05 ± 2.79	0.897

Cronbach’s α = 0.903 CI 0.881–0.916) test–retest reliability: r = 0.810 BIT-C-11, Chinese version of the BIT questionnaire with 11 items; α, Cronbach’s α without item, ICC, intraclass correlation coefficient.

### 3.2 Validity analysis

#### 3.2.1 Content validity

Six diabetologists rated the BIT-C-11’s content validity. The I-CVI for all 11 items was 1.0, all higher than 0.78. The S-CVI/Ave was 1.0 higher than 0.9. They all meet the criterion of content validity (24, 25). The BIT-C-11 has good content validity.

#### 3.2.2 Construct validity

We conducted the EFA on the BIT-C-11 using the PCA combined with the Varimax orthogonal rotation method. The KMO value was equal to 0.830 ≥ 0.7. The difference in the Bartlett’s test was statistically significant (p < 0.01). The chi-square value was equal to 3,131.231. The results demonstrated that the BIT-C-11 was suitable for factor analysis (24, 36).

**Figure 1:**
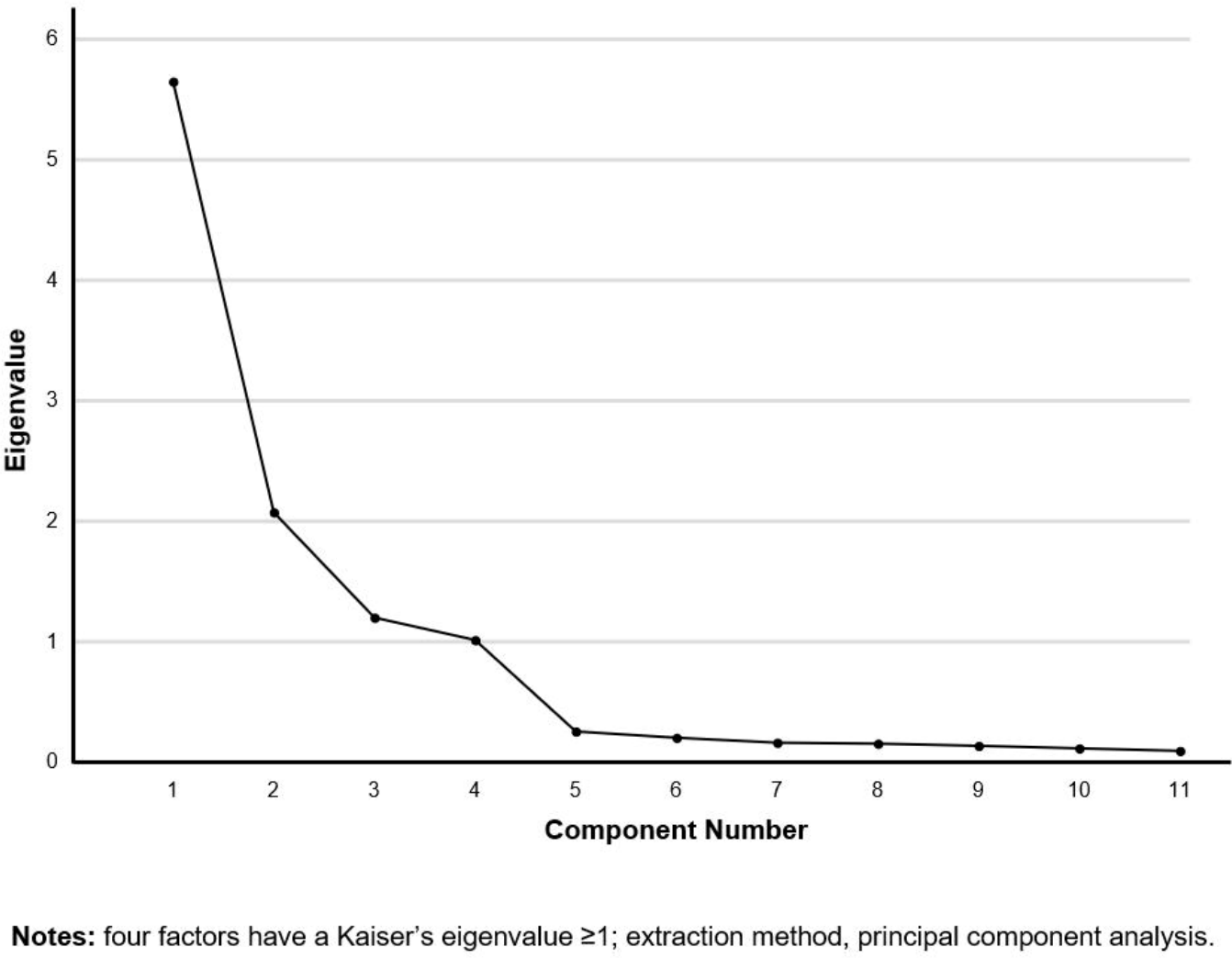
Scree plot.

Three items in Factor 1 are consistent with the BIT’s “fear of injection and self-testing” factor. We named Factor 1 “fear of injection and self-testing” as well. The three items in Factor 2 are consistent with those in the “expected hardship from insulin treatment” factor of the BIT, so we named Factor 2 “expected hardship from insulin treatment.” Factor 3 contains the BIT’s three items of the “stigmatization by insulin injections” factor. Therefore, we also named Factor 3 “stigmatization by insulin injections.” Factor 4 has two items corresponding to the two items in the BIT’s “fear of hypoglycemia” factor. We named Factor 4 “fear of hypoglycemia” (**Table 3**). The scree test also supported extracting four factors (**Figure 1**).

**Figure 2:**
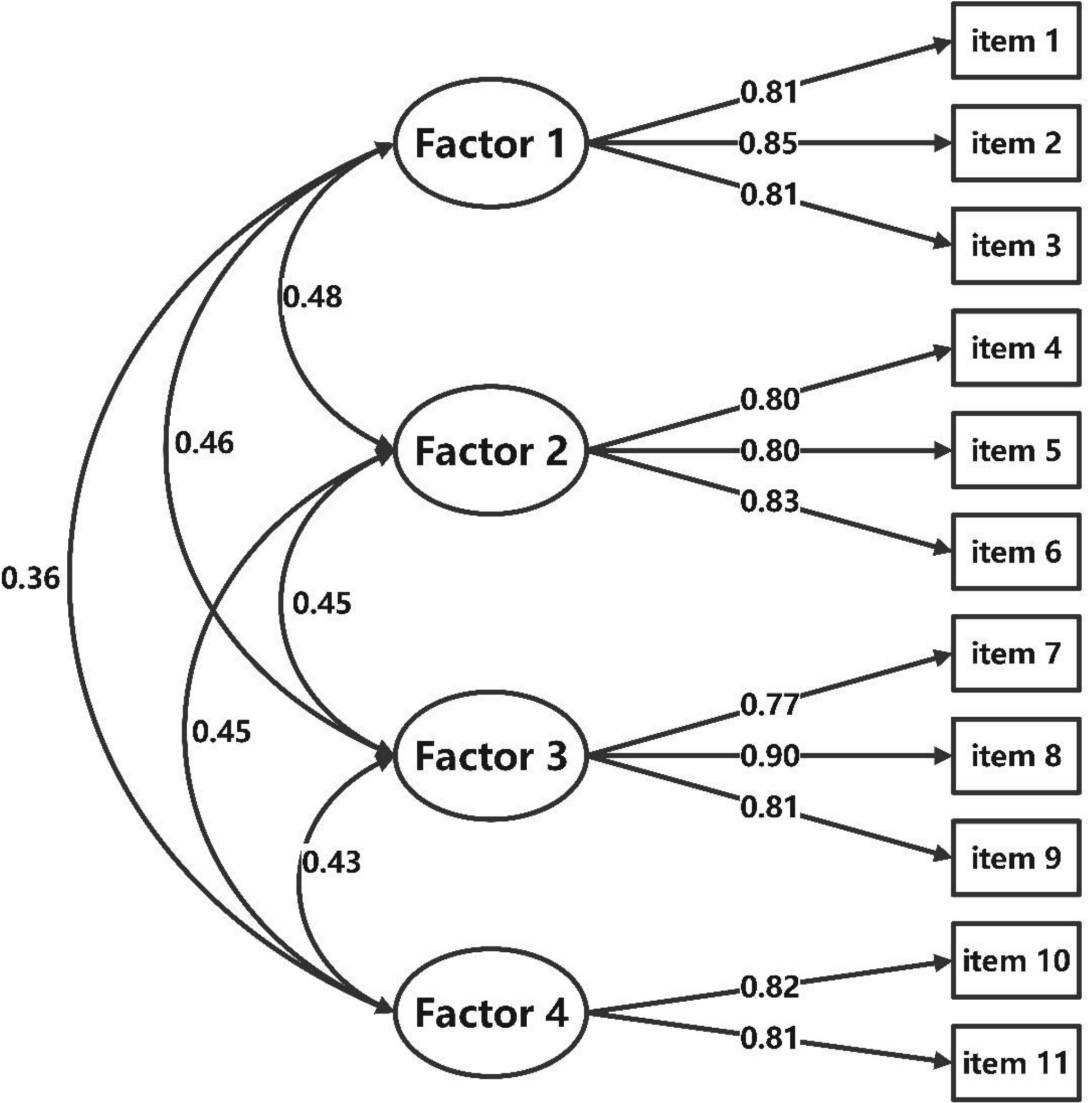
Path diagram for BIT-C-11’s first-order CFA. BIT-C-11, Chinese version of the BIT questionnaire with 11 items; CFA, confirmatory factor analysis; Factor 1, fear of injection and self-testing factor; Factor 2, expected hardship from insulin treatment factor; Factor 3, stigmatization by insulin injections factor; Factor 4, fear of hypoglycemia factor.

**Figure 3:**
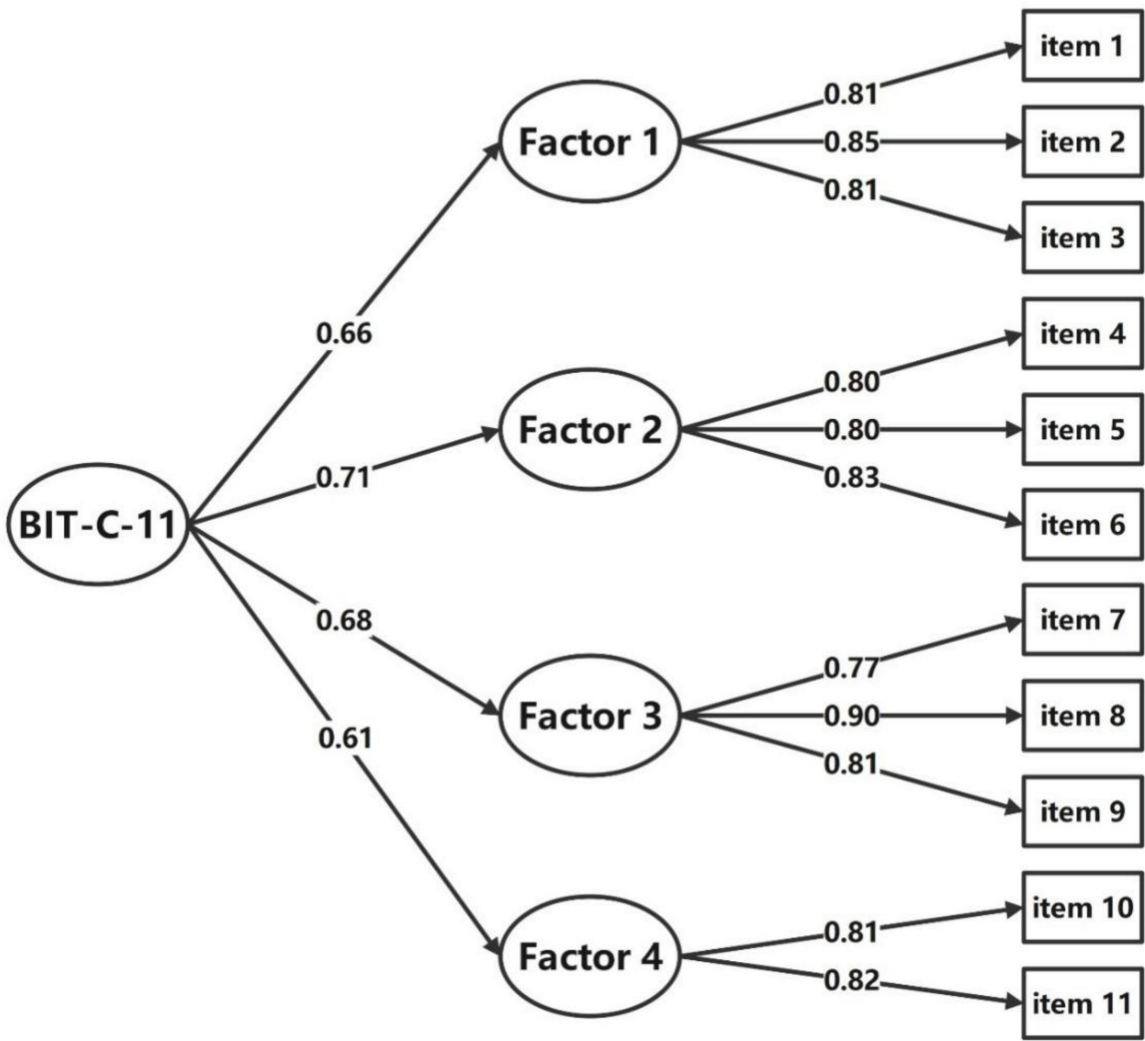
Path diagram for BIT-C-11’s second-order CFA. BIT-C-11, Chinese version of the BIT questionnaire with 11 items; CFA, confirmatory factor analysis; Factor 1, fear of injection and self-testing factor; Factor 2, expected hardship from insulin treatment factor; Factor 3, stigmatization by insulin injections factor; Factor 4, fear of hypoglycemia factor.

Results of the first-order CFA confirmed the structure of the BIT-C-11 with a good model fit with CMIN/DF = 1.311 < 5; GFI = 0.954, CFI = 0.961, TLI = 0.944, their values all >0.9; the SRMR = 0.032 < 0.05, and the RMSEA = 0.040 < 0.05. Standardized factor loadings ranged between 0.77 and 0.90 (**Figure 2**). The second-order CFA of the BIT-C-11 confirmed a good model fit with CMIN/DF = 1.104 < 5; GFI = 0.960, CFI = 0.982, TLI = 0.975; the SRMR = 0.033 < 0.05, and the RMSEA = 0.039 < 0.05. Standardized factor loadings ranged between 0.77 and 0.90 (**Figure 3**), so creating a total score of the BIT-C-11 is appropriate.

#### 3.2.3 Criterion-related validity

A negative correlation exists between the BIT-C-11 total score and the GAS-C total score, with a Pearson correlation coefficient of 0.598, p < 0.001. The criterion-related validity of the BIT-C-11 is acceptable (32).

### 3.3 Reliability analysis

The Cronbach’s α coefficients of the BIT-C-11 and its four factors were 0.903, 0.952, 0.927, 0.938, and 0.917. They are all >0.7 (p < 0.001). The Cronbach’s α values of the BIT-C-11 and its four factors are appropriate (16). The BIT-C-11’s ICC was 0.899 (95% CI 0.881–0.916). The ICC values for Factor 1, Factor 2, Factor 3, and Factor 4 were 0.951 (95% CI 0.940–0.960), 0.921 (95% CI 0.897–0.938), 0.937 (95% CI 0.923–0.949), and 0.915 (95% CI 0.893–0.933), respectively. All of which were >0.75 and met Cicchetti’s criteria for good (28). The correlation coefficient r of the test–retest reliability of the BIT-C-11, Factor 1, Factor 2, Factor 3, and Factor 4 was 0.810, 0.794, 0.756, 0.778, and 0.757, respectively. They are all >0.4. The test–retest reliability of BIT-C-11 and its four factors is acceptable (34) (**Tables 4**, **5**).

**Table 5:** Reliability of the BIT-C-11’s factors.

Factors and items of the BIT-C-11	α
Factor 1: fear of injection and self-testing	
1. I am afraid of the pain when injecting insulin.	0.943
2. Besides the pain, I am afraid of injections.	0.930
3. I am afraid of the pain during regular blood-sugar checks.	0.913
Cronbach’s α=0.952	
ICC: 0.952 (95% CI 0.940-0.960)	
test-retest reliability: r=0.794	
Factor 2: “expected hardship from insulin treatment”	
4. I just don’t have enough time for regular doses of insulin.	0.919
5. I can’t pay as close attention to my diet as insulin treatment requires.	0.905
6. I can’t organize my day as carefully as insulin treatment requires.	0.859
Cronbach’s α=0.927	
ICC: 0.921 (95% CI 0.897-0.938)	
test-retest reliability: r=0.756	
Factor 3: stigmatization by insulin injections	
7. Injections in public are embarrassing to me. Pills are more discreet.	0.925
8. Regular insulin treatment causes feelings of dependence.	0.898
9. When people inject insulin, it makes them feel like drug addicts.	0.905
Cronbach’s α=0.938	
ICC: 0.937 (95% CI 0.923-0.949)	
test-retest reliability: r=0.778	
Factor 4: fear of hypoglycemia	
10. An insulin overdose can lead to extremely low blood-sugar levels (“hypoglycemia”). I am afraid of the unpleasant accompanying symptoms.	*
11. An insulin overdose can lead to extremely low blood-sugar levels (“hypoglycemia”). I have concerns about possible permanent damage to my health	*
Cronbach’s α=0.917 ICC: 0.915 (95% CI 0.893-0.933) test-retest reliability: r=0.757

BIT-C-11, Chinese version of the BIT questionnaire with 11 items; α, Cronbach’s α without item; *, Not applicable because scale contains only two items.

### 3.4 Ceiling effect and floor effect

There were no subjects who achieved the maximum total score of 110. Two subjects achieved the lowest total score of 11, 0.7% of all people. They were all below the criteria of 15% for both the ceiling and floor effects. No subject response bias was observed in the current study (35).

## 4 Discussion

Chinese patients with T2D generally have poor insulin adherence (5). It is urgent to assess the psychological resistance to insulin therapy in Chinese patients with T2D using a standardized scale. Our revised BIT-C-11 has relatively good psychometric characteristics and can be used to assess the psychological resistance to insulin therapy in middle-aged and elderly Chinese patients with T2D.

It is interesting to note that unlike Petrak’s original BIT, which contains five factors, the BIT-C-11 does not include the three reverse score items in the expectations regarding positive insulin-related outcomes factor of the original BIT. The reason for such differences may be due to differences in sample selection.

Petrak et al. (6) selected insulin-naive patients to develop the original BIT, who had no experience of improved health due to using insulin before.

In this study, we selected patients with T2D who were already using insulin as the study subjects. There may be differences in the content of psychological resistance to insulin therapy between patients with T2D treated with insulin and those with T2D not using insulin. Suppose we conduct an in-depth study of patients with T2D psychological resistance to insulin treatment in the future; it might be necessary to classify the study subjects in order to draw more scientific and accurate conclusions.

Another possible explanation is cultural differences. Several studies have suggested cultural differences in psychological insulin resistance, such as, for example, some studies showing ethnic differences in the causes of psychological insulin resistance (37, 38). Among Asian patients with diabetes, especially in China, there is a greater fear of injections and more incredible difficulties in using insulin than Western patients (39, 40). A 2015 study showed that psychosocial factors (rather than the presence of comorbidities) play a more critical role in determining PIR in the Chinese population (41). These studies suggest the influence of cultural differences, but the exact mechanisms are unclear, and more research needs to be done to elucidate them.

Petrak found that patients who opt for oral medications report significantly higher barriers to insulin therapy than those willing to use subcutaneous insulin. The original BIT has an apparent predictive validity for patients’ psychological resistance to insulin therapy (6). However, our subjects were patients who already used insulin injections, so we analyzed the BIT-C-11’s concurrent validity instead of its predictive validity. The GAS is a commonly used scale to assess general adherence in patients with chronic diseases (17). We found a negative correlation between the BIT-C-11 and the GAS in this study. A correlation coefficient of 0.582 > 0.4 means that the criterion-related validity of the BIT-C-11 is acceptable (32). It implies that the better the T2D patient’s general adherence to his or her physician’s recommendations, the lower is his or her psychological resistance to insulin treatment may be. General adherence encompasses many aspects of prevention, treatment, and patient health care. Adherence to insulin treatment is only a part of general adherence, which may explain why the GAS and the BIT-C-11 are related, but the correlation is not too high.

The Cronbach’s α value for the BIT-C-11 was 0.903, and the Cronbach’s α value in the original BIT was 0.78 (6). Both the original BIT and the BIT-C-11 and their subscales have relatively good internal consistency reliability. Since the subjects of these two studies differed, there was little comparability between their α values.

Petrak et al. (6) did not report the test–retest reliability of the original BIT. The correlation coefficient r of the BIT-C-11’s test–retest reliability was 0.810 after 4 weeks, which indicates that the stability of the BIT-C-11 is acceptable (34). Since patients with T2D need to use insulin for a long time, it is possible that some patients’ psychological resistance to insulin therapy decreases over time and become more receptive to insulin therapy. However, due to certain specific events, some patients already on insulin therapy may temporarily increase psychological resistance to insulin therapy and may become reluctant to receive insulin therapy for some time. Therefore, unlike personality traits that remain stable over time (42), we speculate that psychological resistance to insulin treatment is a psychological state subject to change by various factors. This changeability might be the basis for our intervention for psychological resistance to insulin treatment in patients with T2D. Further research is needed to identify the factors influencing psychological resistance to insulin therapy and develop specific and effective interventions to address these influences. However, this opinion needs to be supported by more research data in the future.

## 5 Strengths

The BIT is a valuable research tool to assess patients with T2D’s psychological resistance to insulin treatment, but to our knowledge, the BIT-C-11 is the first revised Chinese version of the BIT. The number of subjects in this study met the sample size requirements for a revised scale study. The study’s objectives and the inclusion and exclusion criteria of the subjects were clear in this study. There were no missing data in the survey due to the efforts of the investigators. The BIT-C-11 has relatively good reliability and validity, and the CFA verified its structure. All of the above are the strengths of this study.

## 6 Limitations

Although we have successfully revised the Chinese version of the BIT, the age and ethnic group of the subjects in this study lacked sufficient representation. On the one hand, worldwide, the trend of T2D in the younger population has surpassed that of the middle-aged and older population in recent years. We only selected patients with T2D aged 45–74 years for the study; further validation is needed if the BIT-C-11 is to be used in other age groups. We will further expand the subject’s age range to improve these shortcomings in the future.

Due to the study’s funding, considering that the BIT contains only 14 items, which is not a large number, we empirically selected 15 patients with T2D who met the study criteria for the pretest and cognitive interviews. Our judgment was not well grounded in theory. It should be noted that sample sizes >30 may be more scientific in pretesting and cognitive interviewing (43).

Another problem is that Factor 4 contains only two items. According to the theory of scale development, each factor should be composed of at least three variables; otherwise, the factor should be discarded or ignored (44). However, considering that “fear of hypoglycemia” is an essential aspect of psychological resistance to insulin therapy, Factor 4 of the BIT-C-11 already has good validity and reliability. We felt that the structure of the Chinese version of the BIT should be consistent with the original BIT, and the “fear of hypoglycemia” factor of the original BIT only has these two items, so we retained Factor 4.

## 7 Conclusion

We revised the Chinese version of the BIT, which has relatively good reliability and validity. The revised BIT-C-11 is four-dimensional and has a total of 11 items, which can be used to assess the psychological resistance to insulin therapy of middle-aged and elderly urban Han people with T2D who use insulin in China.

## Data availability statement

The raw data supporting the conclusions of this article will be made available by the authors without undue reservation.

## Author contributions

MM: conceptualization, methodology, formal analysis, and writing–original draft. XM: methodology, data curation, and writing–original draft. JC: methodology and writing–original draft. FY: methodology and data curation. SM: writing–review and editing. YZ: writing–review and editing. ZS: conceptualization, methodology, writing–original draft, supervision, and funding acquisition. All authors contributed to the article and approved the submitted version.
